# Protein and peptide profiles of rats’ organs in scorpion envenomation

**DOI:** 10.1016/j.toxrep.2023.05.008

**Published:** 2023-05-15

**Authors:** Valery Gunas, Oleksandr Maievskyi, Nataliia Raksha, Tetiana Vovk, Oleksiy Savchuk, Serhii Shchypanskyi, Igor Gunas

**Affiliations:** a Department of Forensic Medicine and Law, National Pirogov Memorial Medical University, Pyrohova Street, 56, Vinnytsia 21018, Ukraine; b Department of Clinical Medicine, Educational and Scientific Center "Institute of Biology and Medicine" of Taras Shevchenko National University of Kyiv, Hlushkova Avenue, 2, Kyiv 03127, Ukraine; c Department of Biochemistry, Educational and Scientific Center "Institute of Biology and Medicine" of Taras Shevchenko National University of Kyiv, Hlushkova Avenue, 2, Kyiv 03127, Ukraine; d Department of Human anatomy, National Pirogov Memorial Medical University, Pyrohova Street, 56, Vinnytsia 21018, Ukraine

**Keywords:** Scorpionism, Venom, Leiurus genus, Middle-mass molecules

## Abstract

Problem of scorpion envenomation becomes more alarming each year. Main effects of scorpion venom are commonly believed to be related to its neurotoxic properties, yet severe symptoms may also be developed due to the uncontrolled enzymatic activity and formation of various bioactive molecules, including middle-mass molecules (MMMs). MMMs are considered as endogenous intoxication markers, their presence may indicate multiple organ failure. Scorpions, belong to the *Leiurus macroctenus* species, are very dangerous, nevertheless, effects of their venom on protein and peptide composition within the tissues remains unclear. In this work we have focused the attention on changes in protein and MMM levels and peptide composition in various organs during *Leiurus macroctenus* envenomation. The results revealed a decrease in protein level during envenomation as well as a significant increment of MMM_210_ and MMM_254_ levels in all assessed organs. Quantitative and qualitative compositions of various protein and peptide factions were continually changing. All of this may suggest that *Leiurus macroctenus* sting causes considerable destruction of cell microenvironment across all essential organs, providing systemic envenomation. In addition, MMM level increment may indicate endogenous intoxication development. Peptides, formed during envenomation, may possess various bioactive properties, analysis of which constitutes an area of further studies.

## Highlights

• *Leiurus macroctenus* venom promotes alterations in protein quantitative and qualitative composition in most vital organs.
• Decrement of protein levels mostly found in brain (by 16.4 %) and liver (by 14.7 %).
• The most significant changes are observed in 24 h after Leiurus macroctenus venom injection.

## Graphical Abstract

**Figure:**
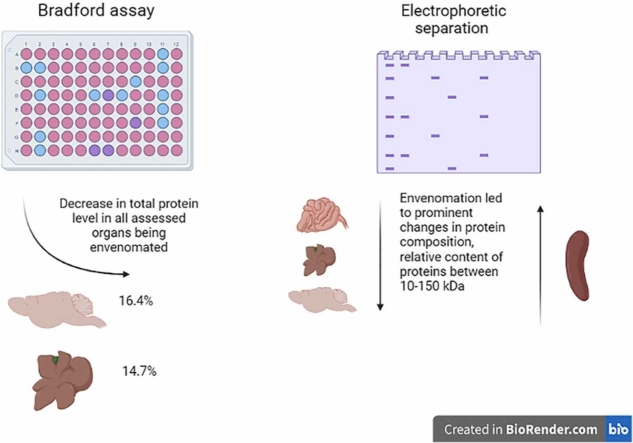

## 1 Introduction

Scorpion envenomation reports number increases from year to year. Uncontrolled expansion of harmful scorpion species as well as urban area enlargement provide more frequent scorpion encountering cases [2], [41]. There are about 1 million scorpion envenomation and nearly 3250 deaths after scorpion sting reports each year [11].

It was recently reported that scorpion sting provides a variety of symptoms the most severe of which are cardio-respiratory dysfunctions [37], hemorrhage [33] and even local tissue necrosis [18]. It’s estimated, that severity and systematicity of scorpion envenomation are closely related to the venom neurotoxicity effects, namely the neuronal excitation and catecholamine release [4], [17], [25]. However, the severe systemic symptoms development may be also associated with increased enzymatic activity within the tissues, which also activates inflammation response [22], [27].

Scorpion venoms are mostly comprised of inorganic salts, nucleotides, amino acids, lipids, enzymes and peptides [29]. Enzymes, present in venom, enhance envenomation process and venom spreading via extracellular matrix destruction [43]. Increased enzymatic activity during envenomation leads to enhanced tissue permeability and provide systemic inflammatory response, since venom toxins can easily spread among all organs and tissues [30]. At the same time, enzymatic biomacromolecule degradation and resulting inflammation development contribute to middle-mass molecules (MMMs) formation [14]. Middle-mass molecules are mixture of molecules with masses don’t exceed 5,000 Da. To MMMs can be referred different tissue destruction products, lipid peroxide compounds and oligopeptides [7]. In turn, MMMs (mostly peptides) are considered as a biochemical marker of multiple organ failure [23] and endogenous intoxication syndrome [28], [31]. Taking into account, that scorpion sting usually provides systemic envenoming [1], [6], MMMs levels may indicate multiple tissue destruction and systemic inflammation development during envenomation.

Scorpions belong to the *Leiurus* genus (also known as “deathstalker”) are well known because of their dangerous venom [17,18], at the same time their venom components possess biomedical properties, attracting scientists’ attention [3,4,12,38]. *Leiurus macroctenus* is a recently identified species [20], that differ from other *Leiurus* species by morphological and morphometric parameters [5].

Unfortunately, little is known about the effect of *Leiurus macroctenus* envenomation on the protein profile in different organs, as well as no information about changes of MMMs levels can be found. This work aims to determine the protein profile and MMMs content in various organs during *Leiurus macroctenus* envenomation. In order to examine given characters, protein content quantification, electrophoretic protein profiling, MMM level analysis and examination of middle-mass peptide qualitative composition was performed.

## 2 Materials and methods

### 2.1 Scorpion collection and maintenance

Ten mature *Leiurus macroctenus* specimens, used in this study, were collected in the wild in Oman, identified by Mark Stockmann and kept in Ibbenbüren private collection (Germany).

Scorpions were kept separately in transparent plastic boxes (10×5x5 cm) filled with sand (Exo Terra "Desert Sand") by 1 cm. Bowls with weekly refilled distilled water served as a water source and were placed in the center of each container. All animal containers were placed under normal conditions (25 °C–35 °C, 50–60 % humidity, natural lighting regime). Appropriate aeration conditions were reached by holes in the boxes. One *Shelfordella lateralis* cockroach were fed to each scorpion once a week, cockroach was taken away in 2 days after feeding in case of food refusal. Boxes were cleaned of cockroach remnants and other pollutions once a month.

### 2.2 Venom collection

Venom collection was performed using Ozkan and Filazi [26], modified by Yaqoob et al. [42]. After appropriate scorpion fixation, electrode was pointed to cephalotorax and telson. To the base of telson for 5 s was applied electric current with intensity of 24 V, while telson’s other edge was pointed to the sample phial. Electrode-scorpion contact number varied from 1 to 10, depending on venom yield. Venom milking procedure was performed every 2 weeks. The collected venom was centrifuged and stored at −20°C.

### 2.3 Venom injection and organ homogenization

Experimental group of 60 albino male rats (180 g ± 3 g) were injected intramuscularly with 0.5 ml venom solution (LD50), previously dissolved in saline solution (0.9 %). Control group, consist of 13 rats, was injected with 0.5 ml saline solution (0.9 %) alone.

Laboratory male rats were raised in the vivarium of the Educational and Scientific Center "Institute of Biology and Medicine" of Taras Shevchenko Kyiv National University. Rats were kept on a standard diet in the conditions of an accredited vivarium in accordance with the "Standard Rules for Organizing, Equipping and Maintaining Experimental Biological Clinics (vivariums)". The following conditions were observed in the room for keeping animals: temperature - 20–24 °C, humidity - 30–70 %, 12-h light day. Rats were fed standard food for laboratory animals. Rats that were selected for the experiment were subjected to a veterinary examination, after which they were divided into groups, weighed, numbered and marked accordingly. The rats were euthanized using the method of carbon dioxide inhalation. The flow rate for 60 % volume displacement per minute was 12.4 liters/min for a cage size of 9" x 17.5" x 8" (W x L x H). Animal execution was followed by organ isolation and homogenization at 1–4 °С. Homogenization was performed using 50 mM Tris-HCl (pH 7.4) buffer with 140 mM NaCl and 1 mM EDTA. Volume of buffer used (in grams) was five times higher than isolated organs’ mass. Obtained crude homogenate centrifugation at 600 g for 15 min with further supernatant collection and its centrifugation at 15,000 g for 15 min were performed to get rid of nuclear and mitochondrial factions. Homogenate aliquots were frozen using liquid nitrogen. Protein contents were measured using Bradford [8].

### 2.4 Eletrophoretic profiling

Electrophoretic separation of protein factions between 10 and 150 kDa was performed in presence of SDS using Laemmli [19]. Concentrations of polyacrylamide in stacking and resolving gels were 4 % and 10 % respectively. Electrophoresis was performed with vertical electrophoresis system (Bio-Rad, USA) by applying electric current of 19 mA and 36 mA to stacking and resolving gels respectively. After separation, protein bands were fixing for 10 min by a mixture of 7.5 % acetic acid and 37.5 % isopropanol. Gels were staining on automatic shaker for 15 min by a mixture of 2.5 % Coomassie Brilliant Blue G-250, 10 % ethanol, 10 % acetic acid and 15 % isopropanol. Excess dye was removed from gels by boiling in 2–8 % acetic acid.

### 2.5 Middle-mass molecules content determination

Faction of MMMs was separated using a method, proposed by Nykolaychyk et al. [24]. The whole procedure was performed on ice, with 15 min breaks between each stage. Tissue homogenate samples were added to equal volumes of 1.2 М HClO_4_ and centifugated for 15 min at 10,000 rpm. To collected supernatant was added 96 % ethanol in ratio of 1:5, then, mixture was centrifugated for 15 min at 10,000 rpm. Optical density of obtained samples was measured at 210 and 254 nm.

### 2.6 Size-exclusion chromatography analysis

Peptide fractions separation and mass determination was performed using Sephadex G15 column (Bio-Rad, USA). Column pre-equilibration was achieved with 0.05 M Tris-HCl (pH 7.4) containing 0.13 M NaCl. Samples were loaded at a flow rate 30 ml/hour. Mass of peptides were determined by calibration curve, calculated using standard mixture containing lysozyme (14.3 kDa), insulin (5.7 kDa), and vitamin B12 (1.35 kDa).

### 2.7 Statistical analysis of results

Values, present in tables are expressed as mean ± SEM. The significance of differences was determined using one-way analysis of variances (ANOVA) performed in GraphPad Prism 9. Differences between groups were considered statistically significant when *p < 0.05.

### 2.8 Ethical approval

All experiments on animals were performed in the compliance with international principles of the European Convention for the protection of vertebrate animals used for experimental and other scientific purposes (Strasbourg, 1986). The study was approved by the Ethical Committee of Taras Shevchenko National University of Kyiv (protocol №2 approved 19.08.2021).

## 3 Results

### 3.1 Protein content quantification

Results of the protein level assay showed a prominent decrease in total protein level in all assessed organs being envenomated (Table 1). The peak of the protein level decrease was observed in 24 h after venom injection. In period from 24th hour to 72nd hour protein levels were increased almost to the control values. Brain and liver protein contents appeared to decrease the most – by 16.4 % and 14.7 % respectively. Protein levels in kidneys, spleen, and intestine have been decreased by 6.5 %, 5.7 % and 5 % respectively; heart and lungs proteins’ contents decreased the least – by 3.5 % and 2.9 % respectively.

**Table 1:** The protein level changes during envenomation.

	Control	1 h	3 h	24 h	72 h
	mg/g of tissue				
Heart	66.56 ± 2.75	65.43 ± 1.24	64.37 ± 0.95	64.22 ± 0.67	65.15 ± 0.76
Spleen	102.15 ± 1.34	98.34 ± 0.98*	97.12 ± 1.13*	96.36 ± 0.94*	100.52 ± 0.94*
Liver	112.23 ± 1.87	98.62 ± 1.25*	97.45 ± 0.56*	95.81 ± 0.76*	109.35 ± 0.46*
Brain	26.8 ± 2.17	23.8 ± 1.11*	23.1 ± 0.95*	22.4 ± 0.34*	25.7 ± 0.33
Lungs	65.01 ± 2.56	65.14 ± 1.15	64.21 ± 1.11	63.13 ± 0.68	64.85 ± 0.63
Intestine	66.19 ± 1.74	65.21 ± 0.89	63.72 ± 0.46*	62.93 ± 0.34*	65.34 ± 0.57
Kidneys	90.01 ± 1.68	87.05 ± 1.23*	86.12 ± 0.95*	84.21 ± 0.75*	88.75 ± 0.84

Protein levels in rat organ homogenates were measured via Bradford assay. BSA was used to prepare a calibration curve. Results are presented as mean ± SEM (n = 5). * p < 0.05 vs. Control

### 3.2 Electrophoretic profiling

In order to evaluate the distribution of protein factions during envenomation, electrophoresis was performed (Table 2). The results showed the alterations in ratio between different protein factions’ levels during envenomation and absolute absence of proteins with molecular weight exceeding 150 kDa before and after venom injection.

**Table 2:** Protein profile of envenomated tissues.

		Control	1 h	3 h	24 h	72 h
	MW, kDa	Relative content, %				
Heart	≥150	0	0	0	0	0
	150–100	0	0	0	0	0
	100–67	5.69	12.56	15.84	13.44	9.73
	67–35	13.26	27.84	28.99	21.52	17.88
	35–10	49.69	39.32	35.88	34.21	43.21
	≤10	31.35	20.28	19.29	30.83	29.18
Spleen	≥150	0	0	0	0	0
	150–100	5.29	9.15	8.75	8.12	6.73
	100–67	0	17.72	24.32	23.77	0
	67–35	23.42	21.84	19.77	15.67	20.74
	35–10	31.6	46.73	35.28	30.95	29.56
	≤10	39.68	4.56	11.88	21.49	42.97
Liver	≥150	0	0	0	0	0
	150–100	4.27	3.56	2.77	3.42	2.99
	100–67	18.86	21.89	29.54	34.23	23.74
	67–35	28.02	42.17	36.54	29.56	30.89
	35–10	29.25	32.38	29.72	31.42	32.64
	≤10	19.61	0	1.43	1.34	9.74
Brain	≥150	0	0	0	0	0
	150–100	2.02	2.34	1.14	0.74	1.64
	100–67	15.32	20.31	11.63	17.83	14.73
	67–35	40.45	35.62	32.17	11.18	32.17
	35–10	42.31	41.73	35.22	44.78	48.88
	≤10	0	0	19.84	25.47	2.56
Lungs	≥150	0	0	0	0	0
	150–100	0	8.9	13.41	16.88	0
	100–67	19.57	17.53	28.58	27.53	19.33
	67–35	39.66	16.82	33.74	25.69	35.19
	35–10	17.35	18.84	16.33	23.36	21.48
	≤10	23.43	8.35	7.89	6.54	24.0
Intestine	≥150	0	0	0	0	0
	150–100	20.12	14.78	12.65	11.87	27.92
	100–67	0	0	3.34	2.64	1.74
	67–35	8.85	19.54	23.99	27.83	14.73
	35–10	29.39	38.43	47.65	48.93	25.85
	≤10	41.64	27.25	12.37	8.73	29.76
Kidneys	≥150	0	0	0	0	0
	150–100	16.65	10.14	0	0	14.86
	100–67	9.46	0	9.86	1.76	7.73
	67–35	22.12	27.89	36.13	40.11	19.56
	35–10	34.15	49.78	50.46	56.83	29.73
	≤10	17.62	12.19	3.55	1.32	28.12


Protein profiles of the tissues were analyzed by electrophoresis in 10 % polyacrylamide gel. The relative content of each protein fractions was evaluated using TotalLab - CLIQS Gel Image Analysis Software and expressed in %.

The levels of a given protein factions were continuously changing. Relative contents of protein faction with molecular weight 100–150 kDa were decreased in liver, brain, intestine almost throughout the whole envenomation period, while in spleen this faction levels were increasing, comparing to the control. At the same time in the lungs, this faction, being absent before envenomation, was observed during first 24 h after venom injection. On the other side, we have observed the complete absence of this protein faction in kidneys from 3rd to 24th hour of envenomation.

Relative content of proteins between 67 and 100 kDa during envenomation was increased in heart, liver and lungs, comparing to the control, furthermore, in spleen and intestine this fraction was absent in control group, but observed during 1–24 h and 3–72 h of envenomation respectively. In the brain changes of this faction’s level were inconstant – it increased in 1 and 24 h after envenomation, but decreased in 3 h and 72 h, comparing to the control. In the kidneys relative content of this faction was majorly decreased.

During envenomation the vast majority of assessed organs had an increment of 35–67 kDa protein relative level, namely heart, liver, intestine and kidneys. In contrast, in spleen, brain and lungs this faction’s levels were decreased.

Also, it was shown, that relative content of proteins between 10 and 35 kDa were predominantly increased in all assessed organs, only in heart this indicator was decreased, comparing to the control.

Envenomation also caused the prominent decrement of < 10 kDa protein relative content in the absolute majority of all assessed organs. Only in brain this faction, being absent in control group, increased its level during 3–72 h after venom injection.

### 3.3 MMM content determination

In order to study the changes of MMM contents during envenomation, MMM faction separation and its further spectrometry analysis was performed. The results of spectrometry measurement at 210 nm (Table 3) and 254 nm (Table 4) showed a significant rise of MMM levels by 53 % and 64 % respectively, compared to the control. It is noticeable that the increment rates were nearly the same in all assessed organs, and the peak of MMM level was observed in 24 h after venom injection.

**Table 3:** The dynamics of MMM_210_ content in tissues during envenomation.

	Control	1 h	3 h	24 h	72 h
	rel. units/g of tissue				
Heart	10.58 ± 0.09	12.75 ± 0.05*	13.56 ± 0.06	16.28 ± 0.03	15.33 ± 0.03*
Spleen	18.02 ± 0.46	21.71 ± 0.04*	23.10 ± 0.07*	27.72 ± 0.02*	26.12 ± 0.03*
Liver	11.05 ± 0.97	13.31 ± 0.09*	14.17 ± 0.05*	17.00 ± 0.03*	16.01 ± 0.02*
Brain	4.15 ± 0.87	5.00 ± 0.11*	5.32 ± 0.05*	6.38 ± 0.02*	6.01 ± 0.01*
Lungs	11.20 ± 1.17	13.49 ± 0.84*	14.36 ± 0.03*	17.23 ± 0.01*	16.23 ± 0.04*
Intestine	19.33 ± 0.13	23.29 ± 0.56*	24.78 ± 0.07*	29.74 ± 0.05*	28.01 ± 0.03*
Kidneys	18.89 ± 1.92	22.76 ± 0.24*	24.22 ± 0.08*	29.06 ± 0.04*	27.38 ± 0.05*

The fractions of middle-mass molecules were isolated from rat organ homogenates by sequential precipitation of proteins and peptides with HCLO4 and ethanol at a final concentration of 0.6 M and 80 %, respectively. The level of the peptides in the MMM fraction was examined spectrophotometrically at a wavelength of 210 nm and expressed as relative units per gram of tissue. Data is presented as mean ± SEM (n = 5). * p < 0.05 vs. Control

**Table 4:** The dynamics of MMM_254_ content in tissues during envenomation.

	Control	1 h	3 h	24 h	72 h
	rel. units/g of tissue				
Heart	1.00 ± 0.01	1.19 ± 0.01*	1.35 ± 0.01*	1.64 ± 0.01*	1.49 ± 0.01*
Spleen	0.44 ± 0.07	0.52 ± 0.02	0.59 ± 0.01*	0.72 ± 0.01*	0.66 ± 0.01*
Liver	0.53 ± 0.03	0.63 ± 0.02*	0.72 ± 0.01*	0.87 ± 0.01*	0.79 ± 0.02*
Brain	0.95 ± 0.20	1.13 ± 0.03	1.28 ± 0.02*	1.56 ± 0.02*	1.42 ± 0.02*
Lungs	0.85 ± 0.08	1.01 ± 0.01*	1.15 ± 0.03*	1.39 ± 0.01*	1.27 ± 0.01*
Intestine	3.54 ± 0.76	4.21 ± 0.01*	4.78 ± 0.03*	5.80 ± 0.01*	5.28 ± 0.02*
Kidneys	4.82 ± 0.85	5.74 ± 0.01	6.51 ± 0.02*	7.90 ± 0.02*	7.19 ± 0.02*

The fractions of middle-mass molecules were isolated from rat organ homogenates by sequential precipitation of proteins and peptides with HCLO4 and ethanol at a final concentration of 0.6 M and 80 %, resp ectively. The level of the non-aromatic sulfur-containing molecules, as well as purine bases and free nucleotides in the MMM fraction, was examined spectrophotometrically at a wavelength of 254 nm and expressed as relative units per gram of tissue. Data is presented as mean ± SEM (n = 5). * p < 0.05 vs. Control

### 3.4 Qualitative composition of MMM peptide component

The evaluation of molecular weight of peptides, present in MMM faction was achieved by size exclusion chromatography. The results showed dynamic changes in middle-mass peptide qualitative composition throughout the envenomation period. Molecular weights of all identified during envenomation peptides are summarized in Table 5.

**Table 5:** Peptide composition during envenomation.

		Control	1 h	3 h	24 h	72 h
	Faction №	Molecular weight, Da				
Heart	1	1611.96	1611.96	1611.96	1611.96	1611.96
	2	1163.87	1163.87	1163.87	1163.87	1163.87
	3	1089.68	1089.68	1089.68	1089.68	1089.68
	4	827.53	827.53	827.53	827.53	827.53
Spleen	1	2114.64	2169.34	2169.34	2169.34	2122.32
	2	1815.02	1871.82	1871.82	1871.82	1843.51
	3	1306.51	1310.22	1310.22	1310.22	1308.33
	4	821.76	1069.87	1069.87	1069.87	834.11
	5			799.52	799.52	
Liver	1	1986.95	1910.47	1910.47	1910.47	1910.47
	2	1378.18	1307.28	1307.28	1307.28	1307.28
	3	1107.39	1067.66	1067.66	1067.66	1067.66
	4		1026.12	1026.12	1026.12	1026.12
	5		798.47	798.47	798.47	798.47
Brain	1	1340.62	1340.62	1340.62	1340.62	1340.62
	2	1252.87	1252.87	1252.87	1252.87	1252.87
	3	1020.38	1020.38	1020.38	1020.38	1020.38
Lungs	1	2147.44	2147.44	2147.44	2147.44	2147.44
	2	1781.80	1781.80	1781.80	1781.80	1781.80
	3	1308.59	1308.59	1308.59	1308.59	1308.59
	4	827.53	827.53	827.53	827.53	827.53
Intestine	1	2355.36	2239.77	2239.77	2239.77	2239.77
	2	1992.65	1751.49	1751.49	1751.49	1751.49
	3	1407.09	1155.24	1155.24	1155.24	1155.24
	4	820.21	989.43	989.43	989.43	989.43
	5		807.43	807.43	807.43	807.43
Kidneys	1	2262.04	2345.81	2345.81	2345.81	2302.34
	2	1489.97	1584.26	1584.26	1584.26	1513.45
	3	1209.45	1265.21	1265.21	1265.21	1232.08
	4	1121.76	978.82	978.82	978.82	1012.44
	5		852.13	855.45	854.23	

The peptide composition of MMM fraction was analyzed by size-exclusion chromatography on Sephadex G15 column. The molecular weight of the peptides was calculated based on a calibration mixture containing lysozyme (14.3 kDa), insulin (5.7 kDa) and vitamin B12 (1.35 kDa).

It was found out, that envenomation had no impact on peptide qualitative composition in heart, brain and lungs, nonetheless, those changes were observed in other organs. In the spleen and kidneys 4 main peptide factions, observed in control group, were replaced with different peptides during 1–24 h of envenomation, which in turn were replaced by peptides with completely different weight. Also, envenomation caused the appearance of additional peptide factions – in spleen during 3–24 h of envenomation and in kidneys during 1–24 h of envenomation.

In liver and intestine changes have been also occurred. In these organs envenomation led to stable changes of peptide qualitative composition, such as replacement of those peptides, being present in control, with another one. Additionally, envenomation provided the appearance of 2 new peptide factions in liver and 1 faction in intestine from 3rd hour after venom injection.

## 4 Discussion

### 4.1 Protein content changes

We have observed the prominent decrease in protein level during envenomation in all assessed organs. It can be explained by the activation of proteases, namely serine proteases and metalloproteases, present in many spider, snake and scorpion venoms [9]. Furthermore, venom components trigger the activation and overexpression of matrix metalloptroteases (MMPs), which, in turn, enhance the damage dealt to extracellular matrix (ECM) [36]. Also, protein level changes occurred in all tissues, suggesting that *Leiurus macroctenus* sting may provide systemic envenoming, which is common for scorpions of *Leiurus* genus [18].

### 4.2 Examination of protein profile

Evaluation of the changes in protein profile during envenomation showed the alterations of ratio between different protein factions in all assessed organs. Levels of protein factions with molecular weight 10–35 kDa, 35–67 kDa, 67–100 kDa and 100–150 kDa had a tendency to rise, while contents of < 10 kDa proteins were decreasing. It can be explained as a consequence of ECM components degradation provided by venom proteases and MMPs. It means that during envenomation these factions to some extent were comprised of the larger molecules’ proteolysis products. To large molecules we refer laminin, collagens, elastin, fibronectin and other ECM components that exceed 200–250 kDa and cannot be observed by electrophoresis method we used. It was previously reported, that some snake [15], [16] and spider [39] venoms can cause ECM degradation and cleavage of its components to numerous fragments with different mass, since such molecules have a huge amount of cleavage sites. Hence, envenomation in our study led to similar effect and obtained increments of the high-mass factions levels are nothing else than a result of matrix proteins’ proteolysis. In contrast, relative contents of < 10 kDa proteins weren’t increasing, which can be explained by electrophoresis resolution limitation. We assume, that the level of proteins and peptides with mass < 10 kDa might be rising, since ECM degradation should have produced a great amount of smaller fragments, but they couldn’t be detected via used electrophoresis method.

The potential consequences of ECM proteins’ degradation could be severe. Under normal conditions ECM components degradation is a common process of ECM turnover [21]. These events are mostly provided by MMPs, and the latters’ activity is strictly regulated by their inhibitors [40].

### 4.3 MMM content during envenomation

We have shown that *Leiurus macroctenus* envenomation affects MMM levels. The peptide component level, measured at 210 nm, as well as non-peptide MMM fraction content, measured at 254 nm, appeared to rise throughout the envenomation period with a peak in 24 h. Taking into account that major protein level decrement was similarly observed in 24 h after venom injection, we may assume that this period of envenomation is the most destructive for cell microenveironment.

The changes in MMM levels are usually associated with intoxication processes and protein metabolism disorders [13], [28]. The variety of molecules which may be involved in autointoxication and can be reffered to MMM is enormous. It includes molecules of normal metabolism, which are present in nonphysiological concentrations; activated zymogens; products of impaired metabolism; products of protein degradation, biogenic amines and many others. The formation of these molecules is mostly related to insufficient micro- and macrocirculation as well as to oxygen transport disruption in tissues. The mechanisms of non-peptide MMMs’ toxicity differ depending on their origin and biochemical properties, yet the obvious reason of their harmful effects lies in their nonphysiological concentrations. One of the most prevalent effect of peptide MMM fraction is cell membrane toxicity. Nevertheless, many peptides (including the protein degradation products) also possess bioactive properties, thus these molecules can imitate hormones, cytokines, neurotransmitters, affecting mitochondrial respriration, DNA and RNA synthesis, glucose metabolism etc. [32]. In addition, increment of MMM levels also may indicate renal dysfunction, since in normal conditions MMMs are metabolized and excreted by kidneys [10]. Taking into account the MMM levels changes in all assessed organs of envenomated rats, we assume that *Leiurus macroctenus* sting might cause systemic endogenous intoxication.

### 4.4 Qualitative composition of MMM peptide component

The results of size exclusion chromatography of MMMs showed presence of new peptide factions during envenomation with mass between 799 Da and 2345 Da. In heart, brain and lungs venom had no impact on peptide qualitative composition, while in other organs the changes were significant. Envenomation led to appearance of completely new peptides, not typical for non-poisoned tissues. Disappearance of factions, being present before venom injection, can be explained by continual proteolytic activity and destruction of those peptides. At the same time, the whole new peptide factions that appeared during envenomation are products of larger proteins’ proteolytic degradation.

In spite of peptide mass evaluation, their potential biological activity remains unrevealed. The properties of the proteolytic ECM degradation products, including MMMs, are unpredictable, since some of them may regulate MMP activity, inflammation development and angiogenesis induction [34], while others may mitigate enzyme activity, disrupt ion transport, suppress the immune system [35]. Hence, identification and bioactive properties characterization of found peptide factions constitute an area of further studies.

In sum, *Leiurus macroctenus* venom promotes significant alterations in protein quantitative and qualitative composition in most vital organs. Examination of protein contents showed a considerable decrement of protein levels in all assessed organs, mostly in brain (by 16.4 %) and liver (by 14.7 %). As it turned out, envenomation led to prominent changes in protein composition, relative content of proteins between 10 and 150 kDa, which were assumed as ECM components’ degradation products, was predominantly increasing. Moreover, MMM level assessment showed a prominent increase in MMM_210_ level by 53 %, in MMM_254_ level by 64 %. The most significant changes were observed in 24 h after venom injection, suggesting that this period of envenomation may be the most destructive and dangerous. In addition, evaluation of peptide quantitative composition showed that some factions with mass between 799 Da and 2345 Da, being absent in control group, appeared during envenomation. These peptides, as well as other ECM degradation products may be harmful for organism, yet their potential bioactive properties analysis create a direction for future studies.

## CRediT authorship contribution statement

Conceptualization: **Valery Gunas**. Data curation: **Tetiana Vovk**. Formal Analysis: **Nataliia Raksha**. Investigation: **Valery Gunas**. Methodology: **Oleksiy Savchuk**. Project administration: **Valery Gunas**. Resources: **Igor Gunas**. Software: **Serhii Shchypanskyi**. Supervision: **Oleksandr Maievskyi**. Validation: **Nataliia Raksha**. Visualization: **Tetiana Vovk**. Writing – original draft: **Oleksiy Savchuk**. Writing – review & editing: **Valery Gunas**.

## Declaration of Competing Interest

The authors declare that they have no known competing financial interests or personal relationships that could have appeared to influence the work reported in this paper.

## Data Availability

The data that has been used is confidential.
